# A New Perspective on the Role of Alterations in Mitochondrial Proteins Involved in ATP Synthesis and Mobilization in Cardiomyopathies

**DOI:** 10.3390/ijms26062768

**Published:** 2025-03-19

**Authors:** Melissa Vázquez-Carrada, María Magdalena Vilchis-Landeros, Héctor Vázquez-Meza, Daniel Uribe-Ramírez, Deyamira Matuz-Mares

**Affiliations:** 1 Institute of Microbiology, Cluster of Excellence on Plant Sciences, Heinrich Heine University Düsseldorf, 40225 Düsseldorf, Germany; m.vazquez-carrada@hhu.de; 2 Departamento de Bioquímica, Facultad de Medicina, Universidad Nacional Autónoma de México, Avenida Universidad 3000, Cd. Universitaria, Coyoacán, Ciudad de México C.P. 04510, Mexico; vilchisl@unam.mx (M.M.V.-L.); hvazquez@bq.unam.mx (H.V.-M.); 3 Departamento de Ingeniería Bioquímica, Escuela Nacional de Ciencias Biológicas, Instituto Politécnico Nacional. Av, Wilfrido Massieu 399, Nueva Industrial Vallejo, Gustavo A. Madero, Ciudad de México C.P. 07738, Mexico; daniel.uriberam@gmail.com

**Keywords:** mitochondria, ATP synthesis, mitochondrial DNA, cardiomyopathies, point mutations

## Abstract

The heart requires a continuous energy supply to sustain its unceasing contraction–relaxation cycle. Mitochondria, a double-membrane organelle, generate approximately 90% of cellular energy as adenosine triphosphate (ATP) through oxidative phosphorylation, utilizing the electrochemical gradient established by the respiratory chain. Mitochondrial function is compromised by damage to mitochondrial DNA, including point mutations, deletions, duplications, or inversions. Additionally, disruptions to proteins associated with mitochondrial membranes regulating metabolic homeostasis can impair the respiratory chain’s efficiency. This results in diminished ATP production and increased generation of reactive oxygen species. This review provides an overview of mutations affecting mitochondrial transporters and proteins involved in mitochondrial energy synthesis, particularly those involved in ATP synthesis and mobilization, and it examines their role in the pathogenesis of specific cardiomyopathies.

## 1. Introduction

The heart is active all the time and requires a continuous energy supply. Hence, the synthesis of adenosine triphosphate (ATP) becomes essential for proper functioning. The heart’s primary metabolic substrate is not glucose but the products derived from the β-oxidation of long-chain fatty acids [1]. Due to the high energy demand, the depletion of cytosolic ATP generates more severe consequences in the heart than in other organs, since its uninterrupted contraction–relaxation cycle [2] requires approximately 6 kg of ATP per day. Most of this ATP is generated in the mitochondria using the electrochemical gradient formed in the respiratory chain from oxidative phosphorylation (OXPHOS) [3].

The mitochondrion is a double-membrane organelle that generates about 90% of cellular energy in the form of ATP in mammalian cells [4]. Mitochondria also play an essential role in several signaling pathways, including the tricarboxylic acid cycle, fatty acid β-oxidation, calcium handling [5], regulation of intrinsic apoptosis, and cell proliferation [6].

Therefore, abnormalities in mitochondrial structure and function are responsible for clinical disorders known as cardiomyopathies: myocardial diseases associated with cardiac dysfunction and conduction defects, sudden death, ischemic and alcoholic cardiomyopathy, and myocarditis [7,8].

Mitochondrial diseases are the most common genetic disorders [9] caused by different mutations in both nuclear (nDNA) and mitochondrial DNA (mtDNA) [10]. Diseases due to mtDNA damage include point mutations and DNA rearrangements, such as deletions, duplications, or inversions, that lead to malfunction of the respiratory chain, resulting in reduced ATP production and an increase in the formation of radical oxygen species (ROS) [7,10,11].

Unlike nDNA mutations, mtDNA mutations are inherited mainly from the mother, which allows for more specific disease behavior monitoring [12]. Currently, no cure has been found, and treatment options for mitochondrial diseases are very limited; only palliative treatments exist to increase the quality of life of patients [13].

On the other hand, the presence of a specialized subcompartment of the endoplasmic reticulum (ER), called the mitochondria-associated ER membrane (MAM), is an intracellular lipid raft-like domain that regulates cellular homeostasis of cholesterol, phospholipids, and calcium and mitochondrial bioenergetics [14,15,16]. When certain mutations occur, the communication between both organelles is altered, which generates or is associated with impaired mitochondrial respiration in several pathologies, including neurodegenerative and cardiac diseases [17,18,19,20].

Alterations in the cellular environment caused by mitochondrial diseases modify the function of the different transporters present in the mitochondria, and, since these transporters regulate the homeostasis of several metabolic pathways and their function is affected by changes in pH and membrane potential, their study is important to understand the role they play in the mentioned pathologies [21]. In this sense, a large number of transporters have been described in mitochondria, including inorganic phosphate transporter, ATP/ADP transporter, citrate/malate antiporter (transports citrate), malate/aspartate shuttle (regulates the passage of 2-oxoglutarate), fumarate/malate or fumarate/inorganic phosphate translocators (transport fumarate), pyruvate/malate, glutamine/malate and d-lactate/malate transporters (transport malate), proline/glutamate translocator and glutamate/OH^−^ transporter (transport proline or glutamate), carnitine–acylcarnitine translocase (translocates fatty acyl-CoA), and ABC transporters [22].

**Figure 1:**
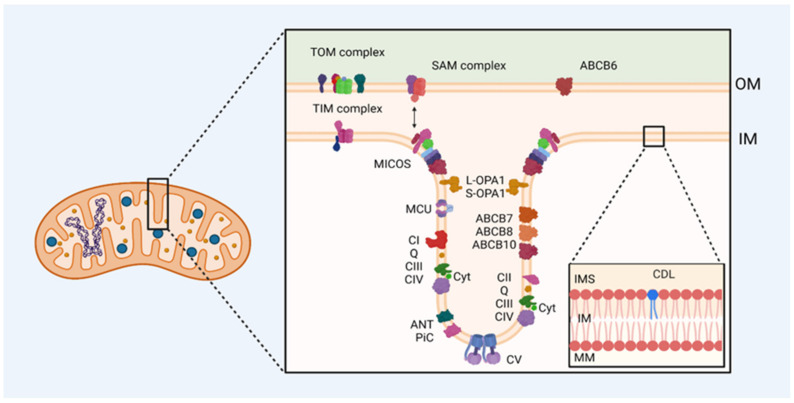
Mitochondrial membrane proteins are involved in ATP synthesis and mobilization in cardiac diseases. Mutations in the genes that encode mitochondrial proteins or damage in them result in many deficiencies in electron transfer and proton-motive force generation altering ATP synthesis and cristae structure. OM: outer membrane, IM: inner membrane, IMS: intermembrane space, MM: mitochondrial matrix, TOM: translocase of the outer mitochondrial membrane complex, TIM: translocase of the inner mitochondrial membrane complex, SAM: sorting and assembly machinery complex, MICOS: mitochondrial contact site and cristae organizing system, MCU: calcium uniporter protein, L-OPA1: dynamin-like GTPase OPA1 (optic atrophy protein 1) long form, S-OPA1: dynamin-like GTPase OPA1 (optic atrophy protein 1) short soluble form, CI: complex I, Q: quinone, CII: complex II, CIII: complex III, CIV: complex IV, Cyt: cytochrome, ANT: ATP/ADP antiporter, PiC: mitochondrial phosphate transporter, CV: complex V, ABC: ABC transporter, CDL: cardiolipin.

This work presents an overview of some proteins associated with mitochondrial membranes, specifically those related to the synthesis and mobilization of ATP (Figure 1), which malfunction due to mutations generating different cardiomyopathies.

## 2. Cardiomyopathies: Generalities

Cardiomyopathies are heart muscle diseases associated with dysfunction, whose severity ranges from asymptomatic forms throughout life to more serious conditions, such as progressive heart failure (HF), arrhythmias, thromboembolism, and sudden cardiac death [23]. They can be classified according to their morphological characteristics in hypertrophic cardiomyopathy (HCM), dilated cardiomyopathy (DCM), arrhythmogenic right-ventricular cardiomyopathy (ARVC), and restrictive cardiomyopathy (RCM) [24,25].

### 2.1. Hypertrophic Cardiomyopathy (HCM)

HCM is characterized by left-ventricular hypertrophy, which is often asymmetric and may involve multiple ventricle regions [25]. Histopathological features of HCM include myocyte disorganization, increased interstitial fibrosis, and abnormal intramural arterioles with thickened walls and a reduced lumen [26]. Mutations in sarcomere proteins have been reported, with approximately 35% of familial HCM attributed to mutations in the gene encoding the β-myosin heavy chain [25,27]. In addition, metabolic alterations and energy impairment are known to be key pathological features of HCM. These alterations include disruptions in mitochondrial oxidative metabolism, suggesting impaired mitochondrial respiratory function [28]. At the cellular level, mutations associated with HCM increase ATP expenditure, leading to a higher energy demand in the heart. Inefficient energy utilization is thought to result from sarcomeric mutations [29].

Symptoms of this type of cardiomyopathy include dyspnea, fatigue, chest pain, palpitations, and presyncopal or syncopal episodes. The pathophysiological mechanisms responsible for these signs include impaired cardiac filling and/or diastolic dysfunction, microvascular dysfunction, and arrhythmias. The most serious complication of HCM is sudden cardiac death, characterized by sudden cardiac collapse or arrest secondary to cardiac arrhythmias [24,30].

### 2.2. Dilated Cardiomyopathy (DCM)

DCM is characterized by enlargement of the left ventricle [31]. This condition is marked by increased heart size, with ventricular walls of nearly normal thickness and varying degrees of fibrosis. Patients often experience progressive HF, with reduced ejection fraction, arrhythmias, and thromboembolic events, as well as fatigue, weakness, and exercise intolerance, all resulting from reduced cardiac output [32,33].

Familiar DCM exhibits genetic heterogeneity, with mutations identified in over 50 genes, which continue to increase [34]. These genes are associated with proteins in sarcomeres, ion channels, cytoskeleton, nuclear envelope, and mitochondria [35]. In addition, allelic heterogeneity exists, as mutations can occur at different positions within many of these genes [32].

### 2.3. Arrhythmogenic Right-Ventricular Cardiomyopathy (ARVC)

This disease is characterized by the progressive fibrofatty replacement of the myocardium in the right ventricle [36]. The clinical presentation is marked by right-ventricular arrhythmias, which can range from premature beats to sustained ventricular fibrillation, potentially leading to sudden death [37]. Disease progression often leads to alterations in the left ventricle, resulting in HF [36]. The replacement of the right-ventricular myocardium by fibrofatty tissue is a degenerative process caused by the progressive death of cardiomyocytes, accompanied by apoptosis and inflammation in the affected area [38]. Mutations in genes encoding desmosomal proteins are the most common genetic defects in these patients. Desmosomes, which are responsible for maintaining cell structure and adhesion, when defective, lead to cell separation, which can induce apoptosis and the death of myocytes, which are subsequently replaced by fibrous and adipose tissue [31].

### 2.4. Restrictive Cardiomyopathy (RCM)

RCM is a rare genetic heart disease characterized by restricted filling of the ventricles and diastolic dysfunction due to heart muscle stiffness, which leads to impaired ventricular relaxation [39]. This disease can manifest at any age, from infancy to adulthood. In infants, the first signs may include failure to gain weight and to thrive, fatigue, and fainting. As the disease progresses, edema, ascites, hepatomegaly, and pulmonary congestion may develop [33]. In adults, RCM typically presents with dyspnea, fatigue, reduced exercise tolerance, arrhythmias, and palpitations [27].

### 2.5. Cardiomyopathies Associated with Mitochondrial Dysfunction

Cardiomyopathies associated with mitochondrial dysfunction are a group of cardiac diseases characterized by alterations in mitochondrial function, essential for cellular energy production. These dysfunctions can result from mutations in mtDNA or nDNA that impair the mitochondria’s ability to generate ATP, thereby affecting myocardial function [40,41,42].

Alcoholic cardiomyopathy is a form of DCM, that refers to a specific disease of the heart muscle developed as a result of chronic and excessive alcohol (ethanol) consumption in individuals with a history of prolonged abuse [43]. Clinical manifestations include ventricular dilation, cardiac hypertrophy, myofibrillar disruption, interstitial fibrosis, and impaired myocardial contractility [44]. The impact of alcohol on the mitochondrial structure of the heart has been demonstrated, showing that alcohol causes mitochondrial enlargement and degeneration of the inner mitochondrial membrane (IM) folds. Recent findings have expanded on this, revealing that chronic alcohol consumption reduces the number of mitochondria without affecting their size and increases mitochondrial fragmentation [45]. Several interrelated mechanisms may include oxidative stress, apoptotic cell death, impaired mitochondrial bioenergetics, mitochondrial stress, and metabolic alterations [46].

Cardiovascular diseases are common in patients with diabetes and contribute significantly to the associated high mortality rates. HF is frequently observed in these people, even in the absence of coronary artery disease or hypertension, and it is referred to as diabetic cardiomyopathy. Evidence suggests that altered myocardial metabolism in diabetes plays a role in contractile dysfunction and ventricular failure [47,48]. The metabolic changes induced by this disease, such as lipotoxicity, glucotoxicity, and impaired insulin signaling, increase oxidative stress, endothelial dysfunction, and inflammation, thereby contributing to both structural and functional damage to the myocardium [49].

The most common cardiac manifestations of mitochondrial diseases are cardiomyopathies [50]. Mitochondrial cardiomyopathies should be considered even in the absence of a diagnosed mitochondrial disease, as they may present solely as a symptom. HCM is the most prevalent form, occurring in 40% of patients; however, mitochondrial cardiomyopathies may also present as non-compaction of the left ventricle or as dilated, histiocytoid, or restrictive cardiomyopathies or may be associated with endocardial fibroelastosis [51].

Various mitochondrial defects have been described in the electron transport chain (ETC) complexes and components of the phosphorylation apparatus in cardiac mitochondria in patients with HF [52].

### 2.6. Other Cardiomyopathies

Other diseases that generate cardiomyopathies have been described, since they modify the cellular physiology of the heart, but it is through other mechanisms and not by some mutation or modification in the mitochondrial proteins. An example is Brugada syndrome, since it can be caused by mutations in the alpha subunit of the voltage-gated Na^+^ channel, encoded by the SCN5A gene. This transmembrane protein is located exclusively in the plasma membrane, rather than in the mitochondria [53]. It is an autosomal hereditary channelopathy that is clinically characterized by episodes of syncope and ventricular arrhythmia that can lead to sudden cardiac death, often in apparently healthy young subjects (≤40 years) [54].

Another example is Chagas disease, which is described as a cardiac condition in which a reduction in cellular ATP levels has been observed. This decrease is mainly due to changes in the activity of creatine kinase isoenzymes, leading to a reduction in phosphocreatine levels and causing varying degrees of myocardial deterioration [55]. Furthermore, a decrease in ATP synthase activity has been reported due to the release of proinflammatory cytokines produced by cells in response to infection caused by the parasite rather than due to mutations in mitochondrial proteins [56,57,58,59].

## 3. Mutations in Mitochondrial Respiratory Chain and Their Relationship with Cardiovascular Diseases

Mitochondria are essential in energetic metabolism because the IM houses the enzyme complexes belonging to the ETC, responsible for generating an electrochemical gradient [60]. The OXPHOS system consists of four respiratory complexes (complex I, reduced nicotinamide adenine dinucleotide (NADH): ubiquinone oxide-reductase; complex II, succinate dehydrogenase; complex III, ubiquinol cytochrome c oxidoreductase; and complex IV, cytochrome c oxidase), the mobile electron transporters coenzyme Q (CoQ) and cytochrome c [61,62,63], and ATP synthase or complex V, which synthesizes ATP [64,65,66].

Mitochondrial DNA is different from that conserved in the cell nucleus; mtDNA is a circular double-stranded molecule, made up of 16,569 base pairs, with 37 genes that encode 13 subunits of four out of five OXPHOS system complexes, the terminal stage of ATP production pathway, 22 transfer ribonucleic acids (tRNAs), and 2 ribosomal ribonucleic acids (rRNAs) [67,68]. From these 13 polypeptides that belong to the ETC, 7 (ND1, 2, 3, 4, 4L, 5, 6) correspond to the subunits of complex I; 1 (cyt b) corresponds to the subunit of complex III; 3 (CO I, II, III) correspond to the subunits of complex IV, and 2 (A, A6L) correspond to the subunits of complex V [69]. These mitochondrial genes provide the basic elements to develop OXPHOS and synthesize mitochondrial proteins. The rest of the subunits of the electron transport chain complexes are encoded in nDNA [70].

Respiratory complexes transfer electrons from NADH and other substrates that originate in different metabolic pathways, such as fatty acid oxidation and the Krebs cycle, to molecular oxygen to form water [64,71]. The electron transfer is coupled to proton translocation from the mitochondrial matrix (MM) to the intermembrane space (IMS), which generates an electrochemical gradient (proton-motive force) across the IM, used by complex V to synthesize ATP [64,65,66]. The transfer of electrons through the respiratory chain complexes allows for oxygen’s reduction to water, but when oxygen reduction is partial, ROS and free radicals are generated [72]. This way, when oxygen captures an electron, the superoxide anion radical (O_2_^.−^) is produced, giving rise to hydrogen peroxide and the hydroxyl radical (∙OH) [73,74,75,76]. In the mitochondrial respiratory chain, electron leakage occurs especially in complexes I, II, and III. It is estimated that 1 to 5% of the oxygen that enters the respiratory chain is converted into hydrogen peroxide, producing free radicals. Moreover, other cellular processes, such as hypoxia, increase ROS production by deregulating the mitochondrial ETC [77,78]. Due to this, mtDNA has a spontaneous mutation rate 10 times higher than nDNA, causing ROS to be continually produced due to the final oxidation of carbon compounds, which can damage mtDNA that lacks histone protection [79,80].

Diseases caused by mitochondrial genome damage share a deficiency in ATP biosynthesis, since all the contained information is directed to the synthesis of OXPHOS system proteins [81]. Even though there are mutations in structural genes of the respiratory complexes, most of the mutations are in tRNAs (Table 1) [82].

The patients who suffer from these conditions only receive palliative treatment to improve their life quality. Currently, there are no effective treatments for mtDNA-mediated disease, so novel investigations into therapy have been sought. One approach entails the selective degradation of pathogenic mtDNA in a heteroplasmic population [104,105], where the mutant mtDNA copy number is specifically diminished, and replication of the spared wild-type mtDNA molecules will result in the cell recovering [106,107].

Unfortunately, only a limited number of disease-causative mutations produce unique restriction sites within mtDNA, and restriction endonucleases are essentially impossible to re-engineer; other methods, also capable of such selective degradation have been developed, and one is the mitochondrially targeted engineered zinc finger-nuclease, a chimeric zinc finger protein with additional mitochondrial targeting sequence and nuclear export signal peptides [107,108], which can be engineered to bind virtually any DNA sequence, overcoming the targeting limitations with mitochondrially targeted restriction endonucleases [109]. Another is the Xanthomonas-derived transcription activator-like effector, which allows for the targeting of both point mutations and mtDNA deletions, offering a hopeful treatment for mitochondrial diseases [110,111]. Although all these technologies appear to be promising, more studies are required to implement them in diary clinics.

### 3.1. Complex I

ROS generated in cardiac injury due to ischemia–reperfusion (IR) are mainly produced by reverse electron transfer (RET) in complex I [112]. During ischemia, mitochondrial succinate accumulates, and upon reperfusion, it is rapidly oxidized, increasing the rate of oxidation of reduced CoQ (CoQH_2_), which increases the proton-motive force, and therefore, electrons are driven through complex I, from CoQH_2_ to flavin mononucleotide (FMN), generating a greater amount of ROS through RET [113,114]. It has been observed when proline is replaced by a leucine (P25L) in the ND6 subunit of complex I, ROS production via RET decreases, with a cardioprotective effect against IR injury [112,114,115]. With the above, it is striking that a mutation generates a favorable response for those who present it, since many of the mutations that occur in these tissues are associated with mitochondrial diseases, affecting the ETC, which, in turn, can cause deficiencies in ATP production and an increase in ROS generation [10,112]. Another example would be the mutation of the NDUFV2 gene (deletion in intron 2), which is associated with the development of HCM [116].

### 3.2. Complex II

Succinate dehydrogenase (SDH, complex II, CII) is an integral membrane complex involved in the Krebs cycle and the respiratory chain [4,117]. Indeed, this complex catalyzes the oxidation of succinate to fumarate, a central step of the citric acid cycle, and reduces FAD to FADH_2_, followed by electron transfer through three Fe/S centers to transform ubiquinone to ubiquinol [118]. Complex II, composed of four subunits (SDHA, B, C, and D) that form two domains, is the only element in the chain that does not pump protons across the membrane and is entirely encoded by nDNA [118]. The hydrophilic head of Complex II (comprising SDHA and SDHB) is essential for succinate oxidation and participates in the electron transfer to ubiquinone. The FAD^+^ cofactor binds covalently to the SDHA subunit, which houses the succinate-binding site and facilitates the transfer of electrons to SDHB. The SDHB subunit contains three iron–sulfur clusters ([2Fe-2S], [4Fe-4S], and [3Fe-4S]). The enzyme’s membrane domain, formed by the SDHC and SDHD subunits, includes a heme b group and two ubiquinone-binding sites [4,118].

### 3.3. Complex III

Ubiquinol cytochrome c oxidoreductase (cytochrome bc1, complex III, CIII) is the central element of the respiratory chain, transferring electrons from CoQH_2_ to cytochrome c [119]. Complex III has a homodimeric structure, and each monomer is composed of eleven different protein subunits. The following three proteins contain four centers involved in redox reactions: (1) cytochrome b with its two CoQ binding sites (Qo or QP, located towards the IMS, and Qi or QN, located towards the matrix) and with heme groups bL and bH; (2) cytochrome c1 with a heme c; and (3) the Rieske protein containing the 2Fe-2S center [120].

Complex III catalyzes the reduction of two cytochrome c molecules by two ubiquinol molecules that are oxidized to ubiquinone in the Qo site, where the two electrons of each ubiquinol molecule follow different paths: one of them is transferred to the 2Fe-2S center of the Rieske protein and the other to heme bL of cytochrome b. In parallel, two H^+^ are released into the IMS [121]. The electron that reaches the 2Fe-2S center of the Rieske protein is transferred to the heme group of cytochrome c1, and from there, it is transferred to the heme group of soluble cytochrome c [119]. 

The removal of the first electron generates a semiquinone in the Qo site that gives up its electron to heme bL of cytochrome b, and from there, it is transferred to heme bH of cytochrome b; the latter transfers the electron to a quinone found at the Qi site, resulting in the production of a semiquinone. To complete the reduction of the semiquinone at the Qi site, the Qo site bifurcation reaction must occur again [122]. In this way, a second ubiquinol molecule is oxidized at the Qo site, another two H^+^ are released to the positive side of the membrane, and a second cytochrome c is reduced through the high-potential chain, while the second electron, transferred through the low-potential chain, eventually reduces the semiquinone to ubiquinol at the Qi site. This is accompanied by the entry of two H^+^ from the matrix [121].

It is necessary to clarify the difference between ROS, radicals, and free radicals. ROS are compounds derived from oxygen, some of which are free radicals and others give rise to some [123]. A radical is a molecule with an unpaired electron in the last orbital and is defined as a free radical when it is found independently of other molecules. For example, ∙OH is derived from O_2_, which is not associated with proteins and can oxidize the membrane phospholipids, initiating lipoperoxidation chain reactions [73]. ROS production takes place in the organelles (mitochondria, lysosomes, peroxisomes, nuclear envelopment) and cytosol of different cell types [74], where NADPH oxidases (NOX) contribute to increasing their concentration, producing O_2_^.−^, nitric oxide synthase (NOS), and NO [124,125]. The greatest ROS production occurs in the mitochondria, mainly due to 11 sites that can generate O_2_^.−^ and H_2_O_2_. Six of these sites operate with the potential of the NADH/NAD^+^ pair (complex I), and the remaining five operate with the ubiquinol/ubiquinone pair (complex III) [72,87].

### 3.4. Complex IV

Cytochrome c oxidase (cytochrome c: oxygen oxidoreductase, complex IV, CIV) catalyzes the final step of the respiratory chain. This complex reduces O_2_ to H_2_O using reduced cytochrome c as an electron donor and consuming H^+^ from the MM. The enzyme has a molecular mass of approximately 200 kDa and, in mammals, is composed of thirteen subunits, ten of which are encoded by nDNA and three by mtDNA (MTcox1, MTcox2, and MTcox3), forming the complex functional core [4].

The electron transfer process involves four metal centers: CuA, heme a, heme a3, and CuB. Electrons donated by reduced cytochrome c are transferred sequentially, one at a time, to the binuclear CuA center. Subsequently, electrons reduce heme a and, subsequently, the binuclear heme a3-CuB center, where O_2_ binding occurs. Four equivalents of reduced cytochrome c are required to generate two molecules of H_2_O, with the concomitant consumption of four H^+^ from the MM [4,126].

### 3.5. Complex V

ATP synthase (F_1_F_0_ ATP synthase, complex V, CV) is a large protein complex located in the IM, where it catalyzes the synthesis of ATP from ADP, Pi, and Mg^2+^, driven by an electrochemical proton gradient generated by the ETC [127]. It consists of two main components: the hydrophilic F_1_ component, which contains the three catalytic binding sites for ATP synthesis, and the F_0_ component, which is embedded in the IM and facilitates the translocation of H^+^ across it [128,129].

Structurally, the F_1_ component is composed of five subunits (α, β, γ, δ, ε) with the stoichiometry α_3_β_3_γδε. Three catalytic sites for ADP binding are located at the αβ interfaces, each adopting one of three conformations: ADP-bound, ATP-bound, or empty. The α and β subunits alternate to form a spherical structure and interact with the γ subunit, which is positioned perpendicular to the membrane plane at the center of the sphere [128,130].

The molecular mechanism of ATP synthesis, proposed by Boyer [128], is based on the rotation of the γ subunit, which induces conformational changes in the β subunits, cycling through the three ATPase states. This rotation is driven by H^+^ translocation through F_0_. With each 120° rotation, the γ subunit contacts a β subunit, forcing it into the empty conformation. Since the three β subunits operate in sequence, a full 360° rotation synthesizes three ATP molecules [128,129].

On the other hand, the hydrophobic F_0_ component channels protons from the IMS through the IM into the MM. This proton-driven motor, known as the oligomycin-sensitive factor, consists of the a, b_2_, and c_10–15_ subunits [131]. The a subunit directly interacts with the c-ring and forms two hydrophilic half-channels that do not span the entire membrane. Protons move from the P-side (positive charge) to the N-side (negative charge) along the electrochemical gradient through a hemichannel formed between the a subunit and the c-ring, inducing rotation [132].

The c subunits form the hydrophobic c-ring, which spans the IM and rotates in response to proton flux through the complex [131,132]. Meanwhile, the b subunit connects F_0_ to F_1_, acting as a static structural element (stator) during rotational catalysis [131,132,133].

Additionally, ATP synthase assembles into long rows of dimers in the IM, which are essential for cristae formation and play a key role in mitochondrial morphogenesis [130,134,135].

## 4. ATP/ADP Antiporter or Carrier (ACC or ANT)

ATP is a product of the breakdown of molecules containing chemical energy (e.g., carbohydrates, proteins, and lipids) [136]. Metabolic processes that utilize ATP convert it back into its precursors: Adenosine diphosphate (ADP) and inorganic phosphate [137]. ATP produced via OXPHOS is transported from the MM to the cytoplasm, while ADP is transported from the cytoplasm to the MM. Over 10% of the IM proteins are ATP-ADP translocases (ANT). This translocase is an antiporter that couples the transport of ATP and ADP molecules [137]. Humans have four different ANT isoforms: ANT1, ANT2, ANT3, and ANT4. ANT1 is predominantly expressed in the mitochondria of cardiac and skeletal muscle tissues.

ANT2 is expressed in regenerative tissues such as the kidney and liver, whereas ANT3 is ubiquitously expressed, and ANT4 can be found in spermatozoa [138,139]. In addition to regulating adenine nucleotide pools, ANT1 increases the sensitivity of the mitochondrial permeability transition pore (MPTP), which appears to be an important early step in certain processes such as apoptosis [140,141].

Alterations in the expression of ANT transporters have been observed in the myocardium of patients with DCM. The pathophysiological effect of impaired ADP/ATP transport leads to altered cardiac energy metabolism and impaired cardiac function in patients with DCM [142]. Mutations in ANT1 cause diseases from cardiomyopathy (A123D) to progressive external ophthalmoplegia (A90D), but the underlying mechanisms are still debated. Defects in ANT1 have also been reported to affect mitophagy, leading to an imbalance in mitochondrial homeostasis and degradation of mitochondria [101,102].

ANT1^−/−^ mice exhibit mitochondrial myopathy characterized by hyperproliferation of abnormal mitochondria, irregular red muscle fibers, and lactic acidosis. This ANT1 defect in mice is also associated with increased ROS, induction of antioxidant enzymes, early accumulation of mtDNA mutations, and downregulation of apoptotic genes [143].

The human A123D mutation in the ANT1 gene, a highly conserved gene, is associated with the accumulation of multiple mtDNA deletions in patient skeletal muscle, leading to loss of function. Patients with this mutation present symptoms of exercise intolerance, muscle pain, a drastic drop in mitochondrial adenine nucleotide translocation in vitro, muscle weakness, or a marked elevation of creatine kinase and HCM [144]. 

ANT1 overexpression has been shown to protect mitochondrial bioenergetics after IR by maintaining basal mitochondrial respiration in a steady state. Complex II activity is increased in hearts with ANT1 overexpression. However, ANT1 does not prevent the loss of necrotic cardiac cells in the infarct zone. Thus, surviving cardiomyocytes in non-infarcted areas compensate for the infarct loss in ANT1 hearts, contributing to less restricted diastolic function after reperfusion [145].

There are no specific treatments to address the conditions caused by these mutations. However, research continues to explore new strategies aimed at improving metabolic and mitochondrial function to slow the progression of these disorders [29,146].

## 5. Mitochondrial Phosphate Transporter (PiC)

The mitochondrial phosphate transporter (PiC) is a mitochondrial solute carrier protein encoded by the SLC25A3 gene in humans. This protein is essential for ATP synthesis, as it serves as the primary mechanism for mitochondrial phosphate import through the IM. This protein has been associated with mitochondrial calcium levels [147]. In addition to its role in energy production, it is suggested that PiC could be involved in cell death as a component or regulator of MPTP [148].

There are two isoforms, PiC-A and PiC-B. PiC-A is limited to cardiac and skeletal muscle, while PiC-B is ubiquitously expressed. PiC-A has a three-fold-higher transport affinity for Pi, whereas PiC-B has a three-fold-higher maximum transport rate [149].

PiC transporter deficiency is characterized by muscle hypotonia, progressive HCM, and elevated plasma lactate levels [144]. Three children with severe neonatal lactic acidosis, HCM, and generalized muscle hypotonia have been reported. One of the children died in infancy, while the other two survived a severe neonatal period. At 9 and 17 years, respectively, they showed exercise intolerance, proximal muscle weakness, nonprogressive HCM, and normal mental development. Muscle tissue biopsies were performed, which revealed a homozygous mutation of the SLC25A3 gene [103]. Subsequently, the clinical characteristics of two new patients with SLC25A3 variants who presented neonatal cardiomyopathy were detailed; one did not show skeletal myopathy or elevated lactate levels. Furthermore, structural modifications in the protein have been described, and it has been recommended to consider SLC25A3 sequencing in patients with isolated cardiomyopathy, even in those without generalized skeletal myopathy or lactic acidosis [150].

Murine models with inducible and specific deletions of the SLC25A3 gene in the heart have been generated. The loss of or reduction in the PiC protein attenuated cardiac IR injury, as well as partially protected cells in culture from Ca^2+^-overload-induced death. On the other hand, long-term deletion of the SLC25A3 gene in the heart resulted in significant hypertrophy, with ventricular dilatation and reduced cardiac function, characteristics that are representative of the cardiomyopathy observed in humans with SLC25A3 mutations [151].

In another study in mice with cardiomyocyte-specific loss of the mitochondrial phosphate transporter (SLC25A3), mitochondrial energy dysfunction was associated with a significant increase in post-translational acetylation and malonylation. These modifications affected a mitochondrial protein interactome, and both modifications were present in the enzyme isocitrate dehydrogenase 2 (IDH2), whose activity was increased. In addition, acetylation of sirtuin 5 (SIRT5), induced by mitochondrial energy dysfunction, was discovered, thus inhibiting its function. Acylations appear to be a mitochondrial response to energy dysfunction and suggest a mechanism by which focal alteration of the energy production machinery may have a broader impact on global mitochondrial function [152].

People with PiC mutations receive treatments that aim to improve quality of life during childhood; some of these treatments include coenzyme Q, levocarnitine, riboflavin, thiamine, creatine, and biotin. Additionally, they are transferred to long-term care centers [150] because there are no specific treatments for this condition, as early death is a common clinical manifestation [153].

## 6. Mitochondrial Contact Site and Cristae-Organizing System (MICOS Complex)

Cristae formation and remodeling are regulated by multiple proteins, including the mitochondrial contact site and cristae-organizing system (MICOS complex), ATP synthase dimers, and the mitochondrial fusion dynamin-like GTPase OPA1 (optic atrophy protein 1) (OPA1). The OMA1 metalloendopeptidase that regulates OPA1 activity [154] has also been implicated in MICOS complex assembly, cristae morphology, and apoptosis. Moreover, solute carrier family member 46 (SLC25A46) is an integral protein of the outer membrane mitochondria (OM) and interacts with mitofilin, OPA1, mitofusin 2 (MFN2), and the MICOS complex to maintain the structure and dynamics of mitochondrial cristae; it also plays a role in lipid homeostasis and phospholipid transfer from the ER [155].

**Figure 2:**
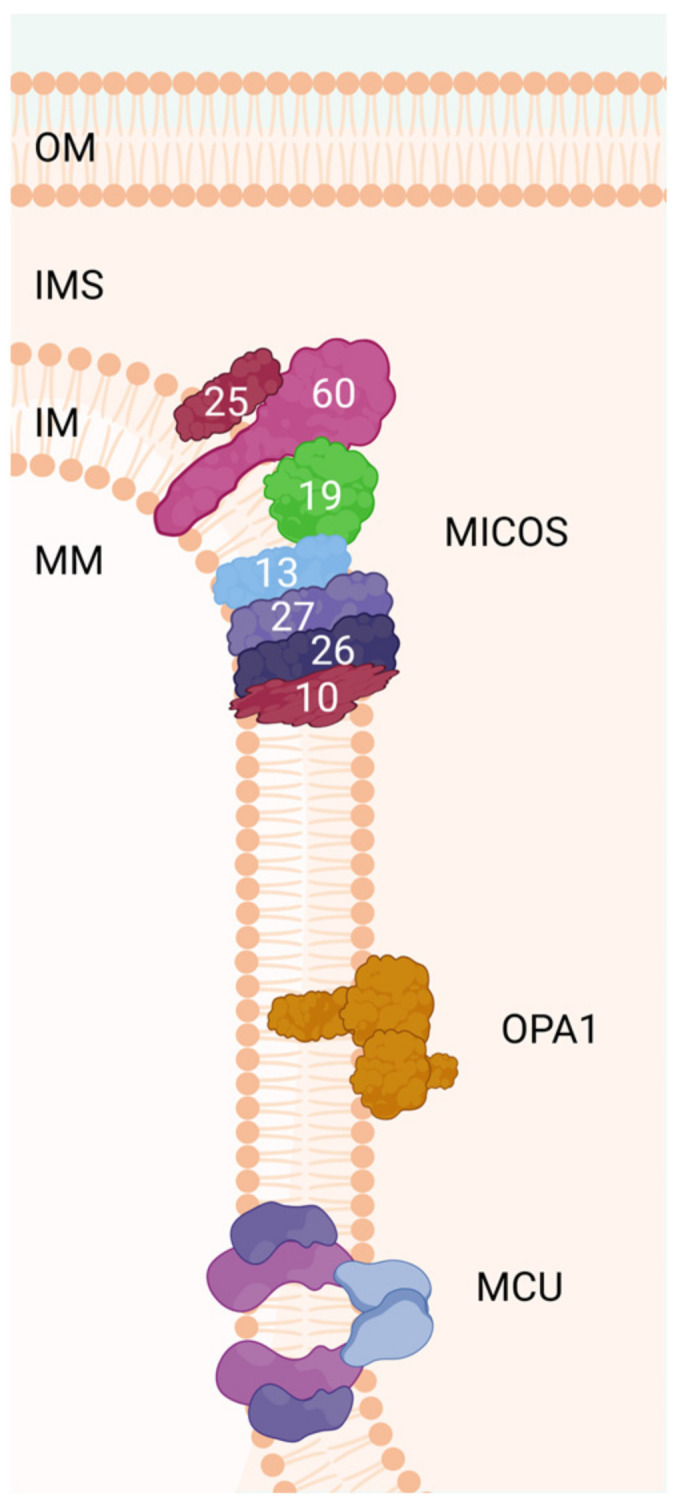
The MICOS complex regulates the dynamics of the inner membrane for the formation of mitochondrial cristae. OPA1 regulates several processes, such as mitochondrial network stability, mitochondrial bioenergy production, and the sequestration of proapoptotic cytochrome c oxidase molecules within the mitochondrial cristae spaces. MCU is a highly conserved protein of the mitochondrial inner membrane that is critical for mitochondrial calcium uptake. The number of each subunit of the complex (human) is indicated in the scheme. OM: outer membrane, IM: inner membrane, IMS: intermembrane space, MM: mitochondrial matrix, OPA1: dynamin-like GTPase OPA1 (optic atrophy protein 1), MCU: calcium uniporter protein.

The MICOS family is a complex of several proteins that link the cristae bases [156]; it was first characterized in yeast. More recently, their human homologs have been identified, and defects in these proteins have been linked to human diseases [157,158]. MICOS subunits are called MicX, where X denotes the molecular weight of the protein (Figure 2). Still, not all MICOS subunits are fully recognized, and 68 are located in the IM and in the internal mitochondrial space (IMS). Additionally, they are organized into two subcomplexes: MIC60 subcomplex (Mic60–Mic19–Mic25) and MIC10 subcomplex (Mic13–Mic10–Mic26–Mic27), joined by Mic13 (Table 2) [157,159,160].

**Figure 3:**
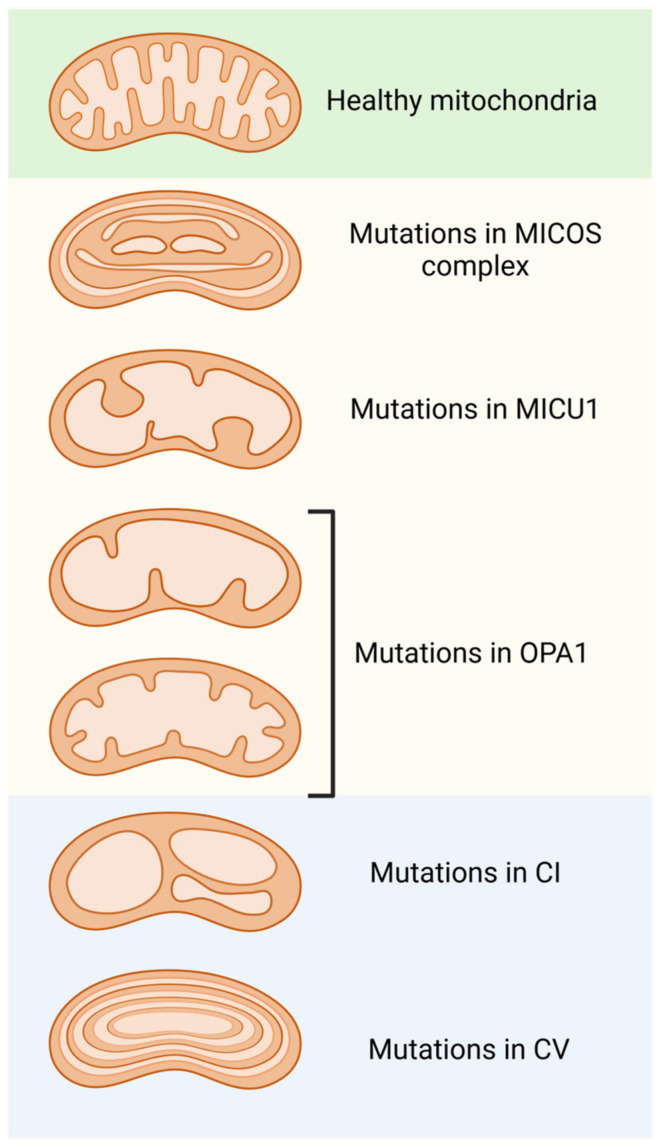
Morphological alterations in mitochondrial cristae are due to modifications or mutations in the various protein complexes found in the inner mitochondrial membrane. Dysfunction of the MICOS complex, MICU1, and OPA1 lead to the disorganization of and a reduction in the number of cristae, as well as enlargement of their junctions. Furthermore, mutations in the components of respiratory complex 1 (C1) result in balloon-like structures in the cristae. Finally, modifications or mutations in ATP synthase (CV) produce concentric onion-ring structures in the cristae.

Yeast MICOS comprises six subunits (Mic10, Mic12, Mic19, Mic26, Mic27, and Mic60), while mammal MICOS also includes an additional subunit (Mic25). The first element of the complex described was the core, Mic60. It is a protein with a large coiled-coil helix domain (60-CC) and a carboxy-terminal mitofilin signature domain (60-MF). Mic60 deletion affects cristae architecture, leading to a variety of abnormal mitochondrial phenotypes: cristae rings, onion-like shapes, cristae detachment from the OM, and cristae leakage (Figure 3) [175].

It has been reported that the deletion of MICOS subunits in yeast leads to the loss of cristae junctions [176]. In addition to this, MICOS assembly controls IM remodeling and the redistribution of cristae junction to mediate cristae formation [177]. The subunits Mic60 and Mic27 were shown to bind to cardiolipin [158]. Also, MIC10 and MIC60 appear to bend the IM to induce negative curvature [156], unlike ATP synthase dimers and oligomers, which contribute to positive curvature at cristae tips [178]. The major component of the MICOS complex, MIC60, is a reported substrate of serine-/threonine-protein mitochondrial kinase 1 (PINK1), a well-described Parkinson’s-disease-associated gene, and it is important for maintaining cristae junctions and appears to control mitochondria-dependent apoptotic cell death, suggesting a possible involvement of this subcomplex in the pathogenesis of Parkinson’s disease (PD) [179]. In addition, MICOS core proteins are in contact with proteins on both IM and OM. Mic19 resides on the IM in front of the IMS and interacts with it.

Furthermore, the MICOS complex is in contact with the OM through the sorting and assembly machinery (SAM), which is important for the insertion of β-barrel membrane proteins into it [179].

The SAM complex is composed of multiple subunits; the core subunit Sam50 binds to two peripheral membrane proteins, Sam35 and Sam37 (Metaxin 1 [MTX1] and Metaxin 2 [MTX2] in mammals). The SAM and MICOS complexes are connected through the axis Sam50–Mic19–Mic60 to mediate the OM and IM contacts by assembling the supercomplex of the mitochondrial intermembrane spatial bridge (MIB) [180].

When the concentration of Sam50 was decreased, mitochondrial cristae formation was absent without affecting the central subunits of MICOS, Mic60 and Mic10, indicating that Sam50 is also essential for cristae formation [109,167]. Depletion of other components, such as Mic19 and Sam37, could impair the SAM-MICOS complex and cristae formation, leading to mitochondrial structure abnormalities and impaired ATP production [181].

On the other hand, a deficiency of Mic60 affects the generation of Δψ and ATP [182,183]. Moreover, mitochondrial respiratory complexes I and IV levels decrease after prolonged Mic60 suppression [168]. Defects in the assembly of respiratory complex IV have also been associated with mutations in Mic14 [184], and the depletion of Mic13 impairs mitochondrial respiration [157]. Furthermore, the deletion of Mic19 alters the levels of two components of respiratory complex IV [167] and leads to significant defects in energy obtention; oxygen consumption and lactate production are dramatically reduced, whilst ATP levels are only moderately affected. Moreover, both the overexpression and downregulation of Mic27 and Mic23/Mic26 showed negative effects on mitochondrial respiration [185].

Additionally, Sam50 depletion decreases the levels of respiratory complexes I and IV and assembly factor RESA1/Coa7 [186], and after a prolonged time, the levels of complex III and ATP synthase are also affected [168]. The observation that the deletion of Sam50 strongly affects the cristae structure but not the levels of MICOS components [168,187] indicates that this effect on respiratory complexes is likely due to changes in the morphology of the IM, which, in turn, could be related to mtDNA stability, since mutations in Mic14 cause a reduction in the number of mitochondrial nucleoids (nucleoids are associated with the IM, are mediated by MICOS, and contain proteins for the replication and repair of mtDNA). At the same time, mtDNA repair under oxidative stress is impaired [184], and Mic60 has also been shown to play a role in mitochondrial nucleoid maintenance and mtDNA transcription [188,189].

### 6.1. Aberrant Mitochondrial Cristae and Related Diseases

Pathogenic variants in MICOS proteins have been linked to heterogeneous human diseases, ranging from early-onset neurodevelopmental disorders to late-onset myopathies, PD, and amyotrophic lateral sclerosis.

Different factors are involved in regulating mitochondrial cristae morphology. Proteins involved in cristae formation can be divided into two categories: members that are located at the junctions of the cristae (MICOS, OPA1, and MICU1) and those that are located at the tip of the cristae (ATP synthase). When these proteins are altered or modified, the morphology of the mitochondrial cristae is radically changed. If proteins located at the cristae junctions are targeted, an aberrant cristae morphology is generated, such as wider cristae junctions, a decreased cristae number, or cristae loss (Figure 2).

On the other hand, ATP synthase dimers are indispensable for membrane curvature at the cristae tip; dysfunction or loss of ATP synthase leads to a concentric onion-ring or balloon-shaped cristae morphology (Figure 2) [190].

Although this classification is not completely strict (for example, onion-shaped cristae also appear in cells with inactivated Mic60), it can be considered a preliminary determination of the cause of the abnormal morphology of mitochondrial cristae in certain disease models. In addition to altering the morphology of the cristae, the structure of the ATP-generating system (OXPHOX) is affected, affecting cell metabolism, cell growth, and division. Mitochondrial cristae characteristics have been shown to correlate with functional changes; in particular, variations in the number and shape of cristae are linked to respiratory efficiency and cell viability [162].

Considering that cellular respiration occurs in the cristae, raising their number may enhance respiratory capacity and ATP synthesis. This hypothesis is supported by the observation that tissues with elevated energy demands, such as muscle tissue, contain mitochondria with a high density of well-developed cristae. In contrast, fibroblast cells, which have lower energy requirements, exhibit fewer cristae [191].

Many proteins involved in shaping mitochondria are directly associated with dysfunctions or perturbations in oxidative phosphorylation. For example, it has been shown that the downregulation of Mic60 and Sam50 impairs the import and assembly of C1orf163/RESA1 and the putative cytochrome c oxidase assembly factor 7, which, in turn, affects the assembly of complex IV [186]. Furthermore, respiratory chain complexes do not function independently; instead, they can form respiratory supercomplexes that enhance the efficiency of the respiratory system. These supercomplexes facilitate both structural and functional interactions among the respiratory chain complexes. The most described types of supercomplexes include I1-III2, I1-III2-IV1, and III2-IV1, among others. The I1-III2-IV1 supercomplex, often called the respirasome, is particularly notable for its ability to complete the entire electron transport process [192,193].

Both in vitro and in vivo studies have demonstrated that abnormalities in cristae structure, resulting from various genetic alterations or apoptotic modifications, influence the assembly and function of respiratory supercomplexes [162]. This process involves several mitochondrial-shaping proteins, including OPA1 and SAM50. Disruptions in cellular metabolism are reflected in compromised ATP production and reduced respiratory efficiency [168,194].

An abnormal morphology of mitochondrial cristae is also observed in some diseases, such as PD, Leigh syndrome, or dominant optic atrophy (DOA), because the proteins that regulate normal functions in the architecture of the cristae are affected, generating or maintaining the pathological processes [195,196].

PD is a progressive neurodegenerative disorder that affects both peripheral organs and the central nervous system. The PARK gene family encodes several proteins, including α-synuclein (α-syn), LRRK2, VPS35, Parkin, PINK1, and DJ1, whose mutations lead to monogenic forms of PD and are therefore critical to the disease’s pathogenesis [197]. PINK1 and Parkin function within the same pathway to regulate mitochondrial quality control [198]. Additionally, Mic60, the core subunit of the mitochondrial contact site and cristae-organizing system (MICOS) has been shown to interact with PINK1, highlighting its significant role in PD [199].

On the other hand, PINK1-mediated Mic60 phosphorylation is crucial for cristae architecture in neuropil and muscle in Drosophila and human cells [200]. Furthermore, several Mic60-coding variants were expressed in PD patients, which altered mitochondrial cristae formation [200].

Another condition related to the change in the morphology of mitochondrial cristae is Leigh syndrome, a well-studied and reported mitochondrial disease, as it is the most common heterogeneous neurological disorder that usually begins in childhood [201]. A deficiency in oxidative phosphorylation is considered a trigger for this disease, which arises from mutations in genes encoding subunits and assembly factors of the OXPHOS complex [202]. On the other hand, mitochondria from patients have been observed to carry mutated subunits of ATP synthase, which affect ATP synthase dimerization and lead to abnormal cristae architecture, resulting in a mild form of Leigh syndrome [203,204].

In 2000, mutations in OPA1 were identified as causal factors in dominant optic atrophy (DOA), a neuro-ophthalmic disorder characterized by bilateral degeneration of the optic nerves, typically manifesting within the first decade of life [205]. Beyond its role in DOA, OPA1 has been implicated in essential cellular processes, including mitochondrial fusion, apoptosis, and autophagy [206,207]. In 2006, Frezza et al. demonstrated that OPA1 regulates cristae remodeling during apoptosis independently of its role in mitochondrial fusion. Apoptosis is orchestrated by several proteins, particularly members of the BCL family, such as BAK and BAX, which are regarded as the “guardians” of mitochondria. Their activation represents a crucial event in the intrinsic apoptotic pathway [206].

### 6.2. Other Disorders Related to Abnormal Mitochondrial Cristae

Cytochrome c release is a main event in apoptosis, occurring after the activation of proapoptotic members of the BCL-2 family. Studies have shown that OPA1 regulates the mitochondrial cristae architecture without interfering with BAK and BAX activation, maintaining tight junctions during apoptosis and thereby inhibiting cytochrome c release as a protective mechanism [208]. In response to injury and infection, inflammatory cells—including neutrophils, eosinophils, lymphocytes, plasma cells, and histiocytes—secrete cytokines, growth factors, and degradative enzymes into the extracellular environment [208]. The role of mitochondrial dynamics in inflammatory cells involved in cellular immunity has been well established, particularly in mitochondrial cristae morphology regulation. For instance, T cell activation triggers cristae remodeling, facilitating metabolic reprogramming that governs immune cell metabolism [209].

A Mic13 mutation leads to early-onset fatal liver disease and encephalopathy with cerebellar atrophy. Hyperlactatemia and 3-methylglutaric acid (3MGA) have been reported [171,210,211]. Loss of Mic13 activity causes a subsequent depletion of MIC10 subcomplex subunits with the MIC60 subcomplex [159]. Interestingly, a clinical case with low activities of complexes I, III, and IV and a decreased mtDNA copy number was described [171], supporting the hypothesis that Mic13 might be involved in mtDNA replication, in common with other MICOS components [212]. Based on this, some authors suggest that Mic13 should be classified as a gene causing mtDNA depletion syndrome [171].

On the other hand, deficiency of the Mic26 subunit (APOO, X-linked recessive mitochondrial myopathy, lactic acidosis, cognitive impairment, and autistic features) has been reported to be associated with a variable form of mitochondrial myopathy with lactic acidosis, cognitive impairment and autistic features, or with a lethal mitochondrial disease with a progeria-like phenotype associated with partial agenesis of the corpus callosum, bilateral congenital cataracts, hypothyroidism, and severe immune deficiencies [213]. Studies of respiratory chain enzymes showed no deficiencies or altered breathing [214].

(A) CHCHD10 deficiency (CHCHD10, monoallelic variants, autosomal dominant inheritance, MIM #615903). CHCHD10-related diseases include mtDNA instability disorder, the clinical spectrum of frontotemporal dementia–amyotrophic lateral sclerosis (FTD-ALS), late-onset spinal motor neuropathy (SMAJ), and Charcot–Marie–Tooth disease type 2 (CMT2) [184,215,216].
(B) CHCHD2 deficiency (CHCHD2, monoallelic variants, autosomal dominant inheritance, MIM #616244). CHCHD2 was the first mitochondrial gene reported to cause PD [217]; however, mutations have also been described in Alzheimer’s disease and frontotemporal dementia. As with CHCHD10 defects, a gain-of-function mechanism with misfolded protein toxicity has been suggested as the pathogenetic mechanism of neurodegeneration [216].
(C) SLC25A46 deficiency (SLC25A46, biallelic variants, autosomal-recessive inheritance, MIM #610826). SLC25A46 deficiency causes Leigh syndrome, optic atrophy spectrum disorder (variably associated with PD), severe sensorimotor axonal neuropathy, cerebellar ataxia, and lethal pontocerebellar hypoplasia [218,219].
(D) ATAD3A deficiency (ATAD3, MIM #612316). ATAD3A participates in mtDNA maintenance through the cholesterol metabolic pathway [220]. ATAD3A interacts with the MICOS complex, and its formation is reduced in ATAD3A-knockout mice [221]. This suggests that mtDNA maintenance may be regulated by interacting with the MICOS complex and ATAD3A. The oligomerization of ATAD3 is necessary for nucleoid mobility [222,223]. ATAD3A deficiency has been associated with a syndrome of neurodevelopmental delay with truncal hypotonia, spasticity, and inherited peripheral neuropathy in an AD or AR form (Harel–Yoon syndrome, MIM #617183) and with a syndrome of lethal AR pontocerebellar hypoplasia, hypotonia, and respiratory failure (MIM #618810). Interestingly, the ATAD3A-associated phenotype has shown enhanced type I IFN signaling, although the pathogenetic mechanisms remain to be determined [224].
(E) Transmembrane protein 70 (TMEM70) deficiency (TMEM70, autosomal-recessive, MIM #614052). This is the most frequently reported cause of nuclear-encoded ATP synthase deficiency, resulting in neonatal encephalic cardiomyopathy with lactic acidosis and hyperammonemia [225]. TMEM70 functions to transport the c subunit of ATP synthase from the TIM complex to OXA1L, a protein required for the insertion of integral membrane proteins into the IM [226].
(F) CLPB deficiency (CLPB, SKD3, biallelic variants, 3-methylglutaconic aciduria, type VIIA, autosomal dominant, 3-methylglutaconic aciduria, type VIIB, autosomal-recessive, neutropenia, severe congenital, 9, autosomal dominant, MIM #616254). It causes autosomal-recessive or -dominant 3MGA, variable neurological disease [227], and neutropenia. CLPB is an ATP-driven disaggregase protein that plays a key role in maintaining mitochondrial cristae stability. The presence of 3MGA associated with isolated severe congenital neutropenia, particularly when a distinct mutation is located in the ATP-binding site (position 105), suggests that this disease may represent a link between defects in membrane biosynthesis and cristae remodeling disorders.

Finally, alterations in mitochondrial cristae morphology in eosinophils have been reported to correlate with their developmental stages. During the final stages of eosinophil maturation, the cristae volume decreases, potentially indicating reduced metabolic activity. However, upon migration into tissue microenvironments, the cristae volume may either increase or decrease depending on the disease state. In response to an inflammatory environment, the number of lamellar cristae declines, while the prevalence of mixed cristae increases. These mixed cristae, characterized by a combination of lamellar and tubular structures, are unique to eosinophil mitochondria. Mitochondrial cristae remodeling has been observed across various inflammatory conditions; however, changes in cristae number, volume, and proportion are largely dependent on the specific tissue microenvironment and disease context [228].

## 7. Cardiolipin

Cardiolipin is an IM phospholipid [229] that interacts with ETC proteins for their optimal function and participates in shaping curved-form cristae morphology [172] and the mitochondria fission and fusion process [230].

Defects in its composition and regulation have been detected as the cause of multiple mitochondrial-dysfunction-related diseases. One of the most studied illnesses is Barth syndrome (BTHS), an X-linked pathology caused by a mutation of the Tafazzin gene (TAZ), which encodes a mitochondrial cardiolipin remodeling/maturation acyltransferase [97]; the deacylation of nascent cardiolipin leads to monolysocardiolipin accumulation [96]. The symptoms include myopathies, neutropenia, and fatigue due to mitochondria morphology defects (size and distribution) [231] observed in the heart biopsies from patients [232]. Mutations have been reported in the 11 exons of TAZ, whose manifestations range from the complete absence of the acyltransferase to a decrease in its expression levels, in addition to the unsaturation of their chains [233,234]. Studies with TAZ-knockout mice reveal increased ROS production, abnormal morphology, ultrastructure, and dynamics, and defects in mitochondrial respiratory chain supercomplex assembly [96]. 

Additionally, approximately 28 point mutations on the TAZ protein have been described. According to their function, they can be classified into two categories: mistargeting to the MM and aberrant complex assembly without mislocalization [97]. 

Not only are mitochondrial bioenergetics altered when TAZ is mutated, but a broad metabolic dysregulation in the cell is also observed, especially for the abnormal TCA metabolite levels present in the BTHS due to the inhibition of pyruvate dehydrogenase, which can be re-established by the addition of calcium, resulting in a regular oxygen consumption rate [235].

The link between the TAZ protein and myopathies is poorly understood, so the development of effective treatments is paused, and only symptom-alleviating medicine is available to increase the life quality and expectancy of patients. Some recent studies suggest a new MyoD1-targeted therapy [236], as MyoD1 is a transcription factor involved in muscle development [237], and its exogenous activity can partially re-establish myogenesis when the TAZ protein is mutated [236].

Despite the TAZ protein being the major enzyme involved in cardiolipin remodeling [238], defects on other proteins participating in the cardiolipin maturation pathway have been related to cardiovascular diseases. For example, mutations on the HADHA (hydroxyacyl-CoA dehydrogenase trifunctional multienzyme complex α subunits) and HADHB (hydroxyacyl-CoA dehydrogenase trifunctional multienzyme complex β subunits) genes, which encode the heterotetrametric trifunctional mitochondrial protein (TFP) [98], lead to decreased oxygen consumption in mitochondria [98]. Fatty acid β-oxidation diseases vary in severity and clinical symptoms. Still, they mainly affect the high- energy-demand organs (heart, skeletal muscle, and liver), which contain mature homoacylated cardiolipins [239]; however, during physiological stress, energy should be obtained via fatty acid β-oxidation [98], so cardiomyopathy and arrhythmias, skeletal myopathy, and recurrent rhabdomyolysis and fasting- and stress-induced hypoketotic hypoglycemia are the most common signs [240].

Additionally, during mitochondria dysfunction due to myopathies or ischemic strokes, cardiolipin becomes peroxidated through O_2_^.−^ production; this acts as a signal for cytochrome c release and, consequently, apoptosis [241]. Phosphatidyl glycerophosphate lipid species play a main role in cardiolipid synthesis [242], so it has been demonstrated that an increase in this compound may protect cells against apoptosis [243].

Another illness related to the abnormal composition of cardiolipin is Senger’s syndrome, caused by mutations in the acylglycerol kinase gene. The encoded protein is localized in the IM and synthesizes phosphatidic acid, a precursor of cardiolipin. This deficiency decreases the mitochondrial adenine nucleotide translocator activity in the muscle [99]. Like Barth syndrome, the symptoms can go from mild to extremely severe, including cataracts, hypertrophic cardio- and skeletal myopathies, and lactic acidosis [244].

Finally, the third known disease associated with cardiolipin defects is dilated cardiomyopathy with ataxia (DCMA). This autosomal-recessive monogenic disorder is caused by variants in the DNAJC19 gene, resulting in short versions of the protein [100], which interact with the membrane protein family of prohibitins that act as a structural scaffold related to cardiolipin metabolism [245]; consequently, patients suffer development and conduction defects, ataxia, male genital anomalies, and growth failure [99].

In the severe forms of these illnesses, most patients have a very low life expectancy, yet an effective therapy is not available. Thus, cardiolipin’s ability to maintain CD8+ T cell function is an open new field for developing advanced treatments. During memory T cell differentiation or nutrient stress, when mitochondrial fitness is required, cardiolipin synthesis is essential to preserve the cell immunity response [246]. Beyond activation, glycolysis and respiratory rate increase, and their products work as signals for T cell differentiation [247].

## 8. ABC Transporters

### 8.1. Iron Metabolism Dysregulation in Cardiovascular Diseases

Iron regulates several biological processes, such as oxygen transfer, cell growth, DNA synthesis and repair, metabolism, and respiration. This essential micronutrient is commonly found in two predominant forms in the human body, including ferric iron (Fe^3+^, oxidized form with limited solubility) and ferrous iron (Fe^2+^, absorbable iron) [248]. To maintain iron homeostasis in the body, many proteins are involved in the import, export, and storage of this element [249]. 

As an energy-intensive organ, the heart has strict requirements for iron homeostasis. Under physiological conditions, a portion of extracellular and cytosolic iron can be imported into mitochondria from the cytoplasm through the OM [249]. Mitochondria have well-characterized functions as the major center of iron metabolism, utilization, and accumulation, and it is the only site for heme synthesis and the primary site for iron–sulfur (Fe-S) cluster synthesis [250]. Heme comprises Fe^2+^ and protoporphyrin IX (PPIX), and it is an indispensable cofactor for several key proteins, including catalases, peroxidases, and cytochrome p450. Furthermore, heme is associated with oxygen storage and transport, signal transduction, electron transfer in enzymatic redox reactions, and regulation of gene expression [251]. Fe-S clusters are required for multiple protein functions, including folding, enzymatic reactions, oxygen sensing, and cell viability. Moreover, they are vital for functional electron transfer in the respiratory chain complexes [252].

Altered iron metabolism typically shows iron deficiency (ID) or iron overload (IO), which are closely associated with various cardiovascular diseases [253]. Disruption of iron homeostasis can severely affect mitochondrial function, leading to impaired energy status [249]. Impaired mitochondrial function is one of the underlying mechanisms of ID-induced HF. ID affects complexes I to IV of the respiratory chain, resulting in altered myocardial metabolism, ROS generation, and advanced HF [254]. In patients with HF, anemia, and ID are frequently observed to cause adverse outcomes and devastating symptoms in patients [255,256]. Although ID is not the only cause of anemia in patients with HF, it is a reversible and relatively diagnosable condition. Also, IO causes diastolic dysfunction and cardiomyopathy, promoting macrophage accumulation and fibrosis through oxidative stress and excessive autophagy, all of which contribute to diastolic HF [257]. Fe^2+^ accumulation in the cytosol triggers ROS production through the Fenton reaction, leading to peroxidation of membrane lipids, membrane damage, and cell death of cardiomyocytes by the process known as ferroptosis [258].

There is abundant evidence to suggest that correcting iron metabolism dysregulation is a key therapeutic intervention for cardiovascular disease and that the treatment of both ID and IO could yield positive patient outcomes [259]. To this end, iron chelators have been administered to moderate iron levels in cases of IO, while iron replacement therapy, including iron supplements or parenteral iron, has been used in cases of ID. However, care must be taken to avoid iron toxicity or deficiency and the generation of ROS [260].

As mentioned above, mitochondria are a key organelle in iron metabolism, and many metabolic processes involved in iron homeostasis occur in them. Eukaryotic cells have developed different transport mechanisms to coordinate the movement of iron and iron-related molecules across membranes. Some transport mechanisms involve ATP-binding cassette (ABC) transporters [261]. The loss of function of these transporters has been associated with the development of some cardiovascular diseases, as will be mentioned below. 

### 8.2. Mitochondrial ABC Transporters

Seventeen ATP-binding cassette (ABC) transporters have been identified in the human heart: ABCA2, ABCA3, ABCA5, ABCA6, ABCA8-10, ABCB1, ABCB6, ABCB7, ABCB9, ABCB10, ABCC5, ABCC8, ABCC9, ABCF1, and ABCG2. These transporters are found in the plasma membrane, ER, Golgi apparatus, lysosomes, peroxisomes, and mitochondria [22]. ABC transporters generally couple ATP binding, hydrolysis, and phosphate release to the translocation of various substrates such as vitamins, steroids, lipids, ions, peptides, proteins, polysaccharides, and xenobiotics [262]. Furthermore, some therapeutic drugs used to treat cardiovascular diseases, such as digoxin, statins, carvedilol, aspirin, and clopidogrel, interact with ABC transporters [263].

Moreover, four ABC transporters have been described in mammalian mitochondria: ABCB6, ABCB7, ABCB8, and ABCB10. These transporters play an important role in cardiac metabolism, as they are involved in iron metabolism and Fe/S protein precursor transport [264]. ABCB10 is also associated with protection against oxidative stress and is essential for erythropoiesis and recovery from cardiac ischemia–reperfusion [265]. Heme is known to act as a cofactor for several biochemical pathways such as the tricarboxylic acid cycle, the oxidative phosphorylation process, and nucleotide biosynthesis [261], so mitochondrial ABC transporters have an important role in energy production and cellular protection, regulating the transport of molecules that can affect energy generation and the production of free radicals [266].

The ABCB6 transporter is located in the OM and is crucial in heme biosynthesis [267]. Heme is involved in electron transfer in mitochondria and may regulate catalase activity to protect cells from ROS [268]. Furthermore, ABCB6 deficiency in bone marrow has been associated with accelerated atherosclerosis in mice [269]. 

ABCB7 is a transporter in the IM and regulates heme and Fe-S biosynthesis. The ABCB7 mutation in humans contributes to X-linked sideroblastic anemia [270]. Chronic high pressure results in ABCB7 transporter deficiency, contributing to iron overload, mitochondrial dysfunction, metabolic change, and worsening cardiac function [271]. Furthermore, the ABCB7 transporter is crucial in controlling apoptotic and non-apoptotic cell death. Therefore, intracellular iron homeostasis modification would be a new anticancer strategy [272].

The ABCB8 transporter is involved in the export of iron from the mitochondria to the cytosol and is important for maintaining normal heart function [273]. Furthermore, this transporter forms a complex with the ATP-sensitive mitochondrial K^+^ channel to protect cells from oxidative stress [274]. Deletion of ABCB8 in mice results in iron mitochondria overload, induces ROS production, decreases the activity of the ETC complex, and reduces the activity of cytosolic enzymes such as xanthine oxidase and glycerol-3-phosphate in the heart. Its loss impairs left-ventricular function and leads to cardiomyopathy. On the other hand, ABCB8 facilitates the export of multiple substrates, including K^+^, iron, glutathione (GSH), and doxorubicin from mitochondria. Cell downregulation results in the accumulation of doxorubicin and its metabolites in mitochondria, generating free radicals and oxidative stress [275].

ABCB10 is located on the IM and is highly expressed in erythroid tissues [276]. It is a transporter that regulates the import of iron. Deletion of the ABCB10 gene results in anemia and embryonic lethality in mice [277]. Hearts from ABCB10^+/−^ mice have normal systolic and diastolic functions, unaltered O_2_^.−^/H_2_O_2_ levels, and normal mitochondrial function, but when these animals are subjected to ischemia–reperfusion injury, progressive HF is observed, suggesting an ABCB10 cardioprotective function [278].

Despite the evidence of mitochondrial ABC membrane transporter dysfunction in several heart conditions, these molecules have not been studied as an adjunctive treatment strategy to counteract mitochondrial dysfunction during HF.

## 9. Conclusions

The study of cardiomyopathies has been developed in clinical and experimental fields. The results reported so far conclude that these conditions are associated with the malfunction of some components of the mitochondrial membranes, such as transporters, respiratory complexes, proteins, and/or lipids. Most of these alterations cause a decrease in the production of ATP due to mutations in the genetic material, both nuclear and mitochondrial. Nevertheless, although the biochemical and molecular mechanisms in which cardiomyopathies occur are known, there is still no definite cure for hereditary mitochondrial diseases, so it is only possible to decrease the illness’ progression. Fortunately, new approaches are in development to diminish the population of mutated DNA through genome editing technologies like programmable endonucleases, base editors, and mitochondria target proteins.

With all the information presented in this work, we conclude that it is necessary to implement a screening test system focused on the most prevalent cardiomyopathies in the population to reduce human and economic losses related to late diagnosis, and it is also required to advance the research and clinical translation (progress in preclinical trials and in vivo administration, dosing, safety challenges, production, and distribution) of treatments focused on restoring the proper functioning of mitochondria, which would allow for an increase in the life quality of patients.

## Figures and Tables

**Table 1:** Human mtDNA mutations associated with cardiomyopathies.

Element	Mutated Gene	Mutation Type	Cardiomyopathy	References
Leucine tRNA, lysine tRNA, isoleucine tRNA, glycine tRNA	MT-TL1, MT-TK, MT-TI, and TRG	Point mutation	Hypertrophic cardiomyopathy, mitochondrial encephalopathy, lactic acidosis, stroke-like episodes, and myoclonus epilepsy with ragged-red fibers	[83]
Complex I, subunits 1–6	ND 1–6	mtDNA point mutation (m.3460G>A, m.11778G>A, and m.14484T>C)	Dilated cardiomyopathy and Leber hereditary optic neuropathy	[84]
Complex I, Cytochrome b	Cyt b	mtDNA point mutation (m.14757T>C)	Hypertrophic cardiomyopathy and macrocephaly–capillary malformation	[85]
Complex II subunits 1 and 2	MC2DNI and MC2DNII	Point mutation, variations on the gene	Dilated cardiomyopathy, Leigh syndrome, and encephalopathy	[80,86]
Complex III, subunits 1 and 3	COI and COIII	Point mutation	Dilated cardiomyopathy	[87]
Complex IV assembly factors	COX10, COX15, and TACO1	Point mutation, variations on the gene	Cardiomyopathy, Leigh syndrome, and encephalopathy	[88,89,90]
Complex IV subunits	MTCO2, MTCO3, and NDUFA4	Point mutation, variations on the gene	Hypertrophic cardiomyopathy and Leigh syndrome	[91]
Complex V subunits	MT-ATP6, MT-ATP8, ATP5I, ATP5MJ, ATP5IF1, and ATP6	Point mutation, variations on the gene	Hypertrophic cardiomyopathy, Leigh syndrome, heart failure, and ischemic cardiomyopathy	[92,93,94]
Mitochondrial cardiolipin remodeling/maturation acyltransferase	TAZ (Tafazzin)	Point mutation, variations on the gene with missing base pairs	Barth syndrome	[92,93,94,95,96,97]
Heterotetrametric trifunctional mitochondrial protein (TFP)	HADHA (hydroxyacyl-CoA dehydrogenase trifunctional multienzyme complex α subunits) and HADHB (hydroxyacyl-CoA dehydrogenase trifunctional multienzyme complex β subunits)	Point mutation, variations on the gene with missing base pairs	Fatty acid β-oxidation diseases	[98]
Acylglycerol kinase	AGK	Variations on the gene with missing base pairs	Senger’s syndrome	[99]
Mitochondrial import inner membrane translocase subunit TIM14	DNAJC19	Point mutation variations on the gene with missing base pairs	Dilated cardiomyopathy with ataxia	[100]
ATP/ADP Antiporter (ANT1)	SLC25A4	Variations on the gene with missing base pairs	Hypertrophic cardiomyopathy and dilated cardiomyopathy	[101,102]
Mitochondrial phosphate transporter (PiC)	SLC25A3	Homozygous mutation c.158-9A>G	Progressive hypertrophic cardiomyopathy	[103]

**Table 2:** MICOS proteins and their possible functions.

	Protein Name	Human Gene Name	Proposed MICOS Function	Other Names	References
MIC60 subcomplex	Mic60	*IMMT*	Core subunit required for normal cristae morphology	Mitofilin, Fcj1, Aim28, Fmp13	[161,162,163,164,165]
	Mic19	*CHCHD3*	Homologue of Mic25, responsible for MICOS–MIB stability through N-terminal myristoylation	MINOS3, AIM13	[166,167]
	Mic25	*CHCHD6*	Mic19 homologue with a role in Mic60 stabilization and cristae morphology	CHCM1	[168,169]
Bridging	Mic13	*QIL1*	Bridges and stabilizes the Mic60 and Mic10 subcomplexes to form the mature MICOS	QIl1, C19orf70, MICOS13	[170,171]
MIC10 subcomplex	Mic10	*MICOS10*	The core of the Mic10 subcomplex, oligomers with Mic60 upholding normal cristae morphology	Mio10, MINOS1, Mos1, Mcs10	[161,162,172]
	Mic26	*APOO*	Paralogue of Mic27, stabilizing Mic10 subcomplex and cristae morphology	Mcs29, Mio27, Mos2	[173]
	Mic27	*APOOL*	Paralogue of Mic26, stabilizing Mic10 subcomplex and cristae morphology	Aim37, Mcs27, MOMA-1	[174]
